# Optimising expression and extraction of recombinant proteins in plants

**DOI:** 10.3389/fpls.2022.1074531

**Published:** 2022-12-08

**Authors:** Ryan J. Coates, Mark T. Young, Simon Scofield

**Affiliations:** School of Biosciences, Cardiff University, Cardiff, United Kingdom

**Keywords:** recombinant protein expression, transgenic plants, plant chassis, transformation, protein purification

## Abstract

Recombinant proteins are of paramount importance for research, industrial and medical use. Numerous expression chassis are available for recombinant protein production, and while bacterial and mammalian cell cultures are the most widely used, recent developments have positioned transgenic plant chassis as viable and often preferential options. Plant chassis are easily maintained at low cost, are hugely scalable, and capable of producing large quantities of protein bearing complex post-translational modification. Several protein targets, including antibodies and vaccines against human disease, have been successfully produced in plants, highlighting the significant potential of plant chassis. The aim of this review is to act as a guide to producing recombinant protein in plants, discussing recent progress in the field and summarising the factors that must be considered when utilising plants as recombinant protein expression systems, with a focus on optimising recombinant protein expression at the genetic level, and the subsequent extraction and purification of target proteins, which can lead to substantial improvements in protein stability, yield and purity.

## Introduction

Recombinant proteins (RPs) are proteins produced in heterologous systems, usually used to increase yields relative to the native system from which the protein is derived. Frequently, these proteins are pharmaceuticals, including vaccines and antibodies, which are produced as recombinant proteins in such scales that the biopharmaceutical sector has previously been the largest sector of the pharmaceutical market (Owczarek et al., 2019). Furthermore, protein drugs contributed to approximately 10% of the drug market in 2017 (Usmani et al., 2017). For biopharmaceuticals specifically, plants have already been shown to be a viable expression platform, and have been recently reviewed (Shanmugaraj et al., 2020), especially for the production of antibodies (Malaquias et al., 2021). Traditionally, mammalian cell cultures are the most favourable expression chassis, and between 2014 and 2018 they were used to produce 84% of novel recombinant proteins, while *E. coli* cell cultures were used for production of 8% and *S. cerevisiae* for 6.5% (Walsh, 2018). Broadly speaking, proteins for vaccines are not needed in substantial yields, but monoclonal antibody production and proteins produced for structural studies need high yields, requiring cost-efficient production platforms (Legastelois et al., 2016). However, it is unlikely that one optimal expression chassis exists for all RPs, as they each have different capabilities, and different RPs have various levels of complexity. For example, some proteins have eukaryotic-specific post-translational modifications (PTMs), and some are membranous meaning they have lower abundance relative to cytosolic proteins due to the reduced area that plasma membranes make up in most cells compared to the cytoplasm. Where possible, recombinant membrane proteins are produced in engineered *E. coli* strains (Schlegel et al., 2014). However, for proteins with PTMs, eukaryotic cell cultures including insect and mammalian cell lines are usually used (Hunter et al., 2018).

There is however an emerging potential for plants to be used as RP expression systems. Plant-made antibodies, or plantibodies, have been investigated as early as 1989 (Hiatt et al., 1989), and have had dramatic success in recent years (Malaquias et al., 2021), for example in producing a successful vaccine for the Ebola outbreak (Zhang et al., 2014), demonstrating that the system is suitable for expression of complex proteins with high yields. Moreover, technologies are developing that allow dramatically improved protein yields in plants, such as hyper-translatable viral elements for transgenes (Thuenemann et al., 2013) and commercially viable plant expression tools (Sainsbury et al., 2009). Due to these improvements, plant expression chassis have become a viable alternative to the conventional mammalian and bacterial systems, though further optimisation is still necessary.

The field of plant recombinant protein production has been heavily reviewed in recent years (Schillberg et al., 2019; Schillberg and Finnern, 2021; Schillberg and Spiegel, 2022), with reviews focussing on the advantages of plant RP expression systems, including the low upstream costs (Avesani et al., 2013; Chen and Davis, 2016), high capacity for scalability (Burnett and Burnett, 2019), potential for edible vaccines (Merlin et al., 2014; Kurup and Thomas, 2019), lack of endotoxin production (Fischer and Emans, 2000; Merlin et al., 2014), rapid protein production times that make them particularly suitable for vaccine production (Zhang et al., 2014; Chen and Davis, 2016; Rattanapisit et al., 2020; Makatsa et al., 2021), and the ability to produce complex post-translational modifications (Gomord and Faye, 2004; Millar et al., 2019), summarized in **Table 1**. Glycosylation is particularly important for biopharmaceutical proteins, with engineering efforts made to humanize plant glycosylation and ensure safe immunogenicity (Sethuraman & Stadheim, 2006; Castilho et al., 2010; Montero-Morales & Steinkellner, 2018).

**Table 1:** Comparison of the capabilities of plants, mammals, and bacteria the carry out the 13 most common Eukaryotic PTMs, according to Minguez et al. (2012).

	Plants	Mammals	Bacteria
**Phosphorylation**	Present (Xu et al., 2019)	Present (Minguez et al., 2012)	Present (Garcia-Garcia et al., 2016)
**N-linked glycosylation**	Present (Strasser, 2016)	Present (Minguez et al., 2012)	Present but different and poorly studied (Nothaft and Szymanski, 2013)
**Acetylation**	Present (Boyle et al., 2016)	Present (Minguez et al., 2012)	Present (VanDrisse and Escalante-Semerena, 2019)
**O-linked glycosylation**	Present (Gomord et al., 2010)	Present (Minguez et al., 2012)	Present but different and poorly studied (Nothaft and Szymanski, 2010)
**Ubiquitination**	Present (Miricescu et al., 2018)	Present (Minguez et al., 2012)	Similar machinery to mimic Eukaryotic ubiquitination (Pisano et al., 2018)
**Methylation**	Present (Serre et al., 2018)	Present (Minguez et al., 2012)	Present with differing substrate specificity (Zhang et al., 2017)
**SUMOylation**	Present (Park et al., 2011)	Present (Minguez et al., 2012)	Absent but can be engineered (Iribarren et al., 2015)
**Hydroxylation**	Present (Duruflé et al., 2017)	Present (Minguez et al., 2012)	Present (Szaleniec et al., 2018)
**γ-Carboxylation**	Absent – unique to some animals (Bandyopadhyay et al., 2002)	Present (Minguez et al., 2012)	Absent – unique to some animals (Bandyopadhyay et al., 2002)
**Palmitoylation (S-acylation)**	Present (Zheng et al., 2019)	Present (Minguez et al., 2012)	Absent (Sobocińska et al., 2018)
**Sulfation**	Present (Kaufmann and Sauter, 2019)	Present (Minguez et al., 2012)	Present (Han et al., 2012)
**Nitrosylation**	Present (Feng et al., 2019)	Present (Minguez et al., 2012)	Present (Gusarov and Nudler, 2012)
**C-linked glycosylation (C-mannosylation)**	Absent (Shcherbakova et al., 2017)	Present (Minguez et al., 2012)	Absent (Shcherbakova et al., 2017)

There are several available plant chassis that each have advantages and disadvantages, reviewed in detail by Sabalza et al. (2013) and more recently by (Gerszberg and Hnatuszko-Konka, 2022). Tobacco (*Nicotiana* spp.) is often used for RP production due to its rapid growth time, scalability, large number of seeds, and well-established transgenesis methods (Ma et al., 2003; Chen and Lai, 2015; Burnett and Burnett, 2019; Moon et al., 2019). Furthermore, the most commonly used plant cell culture is the tobacco Bright-Yellow 2 (BY-2) cell line, however, plant cell cultures rarely present any significant advantages over other eukaryotic cell cultures (Schillberg et al., 2019; Karki et al., 2021; Sethi et al., 2021). Legumes have high protein content (Burnett and Burnett, 2019), rapid growth (Abd-Aziz et al., 2020) and several transformable species such as *Mucuna bracteate*, but often show unfavourable heterogenous glycosylation (Abd-Aziz et al., 2020; Debler et al., 2021). Fruit and vegetable crops, including *Lactuca* and *Solanacea* species have been made to produce RPs, with the main advantage that they can be eaten, negating downstream purification costs (Merlin et al., 2014; Kurup and Thomas, 2019; Moon et al., 2019; Shanmugaraj et al., 2021). For these, several tissue-specific promoters are available (Lim et al., 2012; Ding et al., 2022), and researchers have found that growth conditions can dramatically affect protein content (Matsuda et al., 2009). Aquatic plants can be used, in particular *Lemnoideae* or *Wolffia* species, due to their fast growth rates and high yields, but transformation methods are poorly optimised (Khvatkov et al., 2018; Chanroj et al., 2021). Cereals present another promising expression system, recently reviewed (Mirzaee et al., 2022), with the advantage that their seeds have a high protein content and enable RPs to be stably stored (Stöger et al., 2000). Commonly used cereals include maize (Naqvi et al., 2011; Sabalza et al., 2013), barley (Ritala et al., 2008; Magnusdottir et al., 2013; Shanmugaraj et al., 2021), and rice, the latter particularly as cell-cultures (Huang et al., 2002; Kuo et al., 2013; Huang et al., 2015; Liu et al., 2017). Mosses such as *Physcomitrella patens* have had success for RP production (Baur et al., 2005; Gitzinger et al., 2009; Niederkrüger et al., 2014; Reski et al., 2018), particularly for glycosylated biopharmaceuticals (Huether et al., 2005; Decker & Reski, 2007; Reski et al., 2015), where RPs can be secreted into culture medium which dramatically reduces downstream purification costs (Schaaf et al., 2005). In fact, α-galactosidase A produced in moss has been shown to be more effective in treating enzymatic deficiencies in mice than the more conventional agalsidase alfa (Shen et al., 2015), demonstrating the potential superiority of the system. Finally, some newer expression systems are available, including cell-free protein synthesis systems such as the tobacco BY-2 cell-line lysate-derived system ALiCE, and several wheat germ systems, which have had success in producing cytoplasmic and microsomal proteins (Buntru et al., 2014; Harbers, 2014; Buntru et al., 2015). Similarly, plant cell packs utilise porous plant cell aggregates without cultivation media (Rademacher et al., 2019), compatible with well-plates which enable high-throughput rapid construct screening and can be automated to minimise batch variation (Gengenbach et al., 2020).

Plants can also be transformed using a variety of different methods, depending on a researcher’s needs. Stable transformation requires a single transformation event which is stably inherited in progeny of the primary transformant. This can be nuclear transformation, which typically results in moderate expression levels, but can be subject to transcriptional gene silencing through DNA methylation (Gallego‐Bartolomé, 2020). The site of transgene integration in the genome is generally random (Jaganathan et al., 2018), though some targeted transgenesis methods have been developed (Sanagala et al., 2017). Alternatively, plastids can be stably transformed, enabling very high expression levels due to the high plastid copy number within cells (Oey et al., 2009) and desirable biocontainment through primarily maternal inheritance (Ruf et al., 2007; Gerszberg and Hnatuszko-Konka, 2022). However, plastids are unable to produce complex PTMs. Lastly, transient expression is very rapid, taking days, with well-developed methods and high-expression levels and the ability to produce complex PTMs (Chen and Lai, 2015; Chen and Davis, 2016), with the main drawback that transformation needs to be repeated for each batch of RP. **Table 2** provides a range of examples of recombinant proteins that have been expressed in plants.

**Table 2:** Selected examples of recombinant protein production in plants.

Recombinant protein	Host species	Transgenesis Type	Max Reported Yield	Notable Addition(s)	Reference
Alkaline Phosphatase	*N. tabacum*	Stable - nuclear	1.1μg/g DW (3% TSP)	Used phyllosecretion of the protein to simplify purification	(Komarnytsky et al., 2000)
alpha-1-antitrypsin (rAAT)	*Oryza sativa L.* cell culture	Stable - nuclear	247 mg/L (10% TEP)	Used inducible α‐amylase (RAmy3D) promoter	(McDonald et al., 2008)
Anti-toxoplasma IgG	*M. bracteata*	Transient	591.1μg/g FW	Found that older leaves had the highest expression and that this species had 2-fold higher expression than *N. benthamiana*	(Abd-Aziz et al., 2020)
Cry2Aa2 (Bt. Toxin)	*N. benthamiana*	Stable - chloroplastic	45.3% TSP in leaves	Used chloroplast expression	(Cosa et al., 2001)
Green fluorescent protein (GFP)	*N. benthamiana*	Transient – viral vectors	3.7mg/g FW in leaves	Used a double terminator to increase expression by 2-fold Used geminiviral vector for its broad host range Also transformed lettuce tomatoes, eggplants, hot peppers, melons, and orchids	(Yamamoto et al., 2018)
GFP	*N. benthamiana*	Transient – viral vectors	1mg/g FW (30% TSP)	Used novel pEff vector, with p24 silencing suppressor	(Mardanova et al., 2017)
GFP	*N. benthamiana*	Transient – viral vectors	4mg/g FW in leaves	Used magnifection with TMV-vectors (early magnICON system)	(Marillonnet et al., 2005)
Hepatitis B core antigen (HBc)	*N. benthamiana*	Transient- viral vectors	800μg/g FW in leaves	Used BeYDV-derived vector and p19 silencing inhibitor	(Huang et al., 2009)
hGAD65mut	*N. tabacum*	Stable - nuclear	114.3μg/g FW in leaves	Tissue can be eaten – no purification Generation of elite lines took 6 generations of selfing and 3 years	(Avesani et al., 2013)
human granulocyte colony-stimulating factor (hG-CSF)	*Wolffia arrhiza*	Stable - nuclear	35.5μg/g FW (0.194% TSP)	Aquatic plant host with very rapid doubling time and high protein content *Wolffia* may be better than *Lemna* as the former has no root system and can be submerged	(Khvatkov et al., 2018)
Human Growth Hormone (hGH)	*N. benthamiana*	Transient – viral vectors	170μg/g FW in leaves	Used CMV expression vector	(Fujiki et al., 2008)
human interleukin‐6 (hIL6)	*N. benthamiana*	Transient	18.49μg/g FW after purifying	Used a family 3 cellulose‐binding domain affinity tag to enhance purification	(Islam et al., 2018)
Miraculin	*Solanum lycopersicum*	Stable - nuclear	340μg/g FW	Codon optimisation and use of the protein’s native terminator had highest expression	(Hiwasa-Tanase et al., 2010)
Murine IgG	*N. benthamiana*	Transient	147.7mg/g FW	Use of the p19 silencing inhibitor increased expression by 14-fold	(Kuo et al., 2013)
Norwalk Virus Capsid Protein (NVCP)	*N. benthamiana*	Transient – viral vectors	860μg/g FW in leaves	Used the magnICON expression system	(Santi et al., 2008)
thymosin α1 concatemer	*Solanum lycopersicum*	Stable - nuclear	6.098μg/g FW in fruits	Used polygalacturonase promoter for fruit specific expression	(Chen et al., 2009)
β-glucuronidase	*Lemna minor L.*	Stable - nuclear	1.43% TSP	High protein content in Lemna (up to 45% of DW)	(Kozlov et al., 2019)
Human Serum Albumin	*Physcomitrella patens*	Stable - nuclear	0.3 µg/mL	Secreted protein into culture medium	(Baur et al., 2005)
Human glucocerebrosidase	*Nicotiana.* Root Culture	Stable - nuclear	1 µg/g of root	Analysed N-glycosylation patterns	(Naphatsamon et al., 2018)
Influenza haemagglutinin (from H5N1 and H1N1) Virus-like particles	*N. benthamiana*	Transient	50 mg/kg FW	Electron microscopy showed VLPs accumulate in apoplastic indentations of the plasma membrane	(D’Aoust et al., 2008)

Yield abbreviations are: Fresh Weight (FW), Dry Weight (DW), Total Soluble Protein (TSP), Total Extracted Protein (TEP). This table is not an exhaustive list, but rather shows a range of examples of recombinant proteins that have been produced in different plant species, using different methods of transgenesis, and their resulting yields.

While there is extensive literature about RP production in plants, little information is available about optimising expression through genetic manipulation and subsequent protein extraction. Consequently, this review focusses on the optimization of RP expression at the level of transcriptional and translational control, and the subsequent extraction methods employed for RP purification, with the aim of providing a comprehensive summary of the necessary considerations for producing RPs in plants.

## Optimising expression

**Figure 1:**
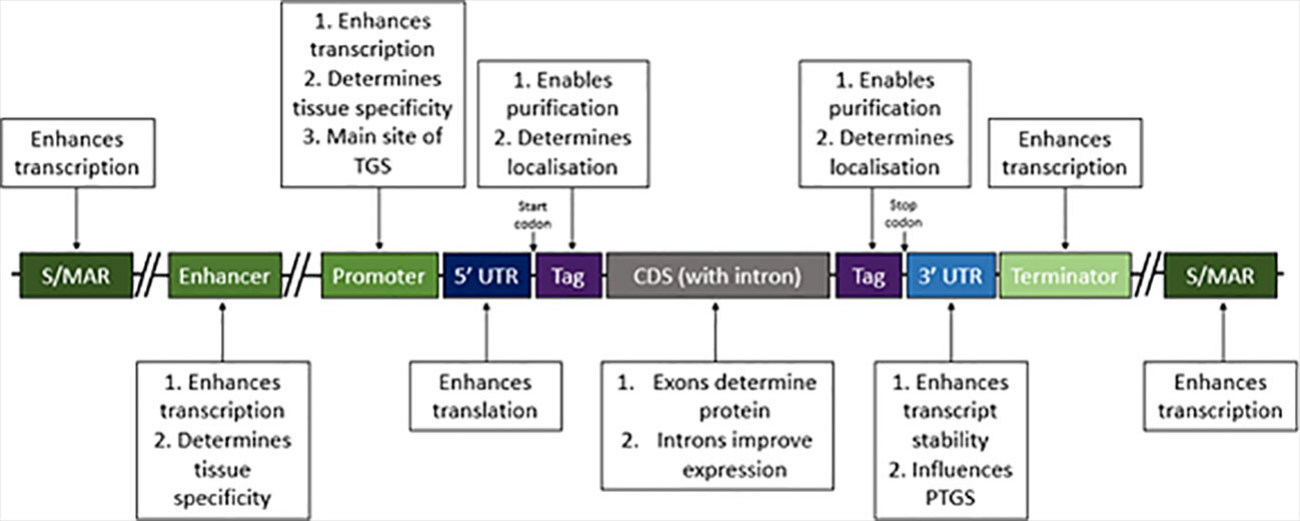
Regulatory DNA elements to consider when making plant RP expression constructs. Some DNA elements are essential in expression constructs, including a promoter, the coding sequence (CDS) and terminator; however many components in expression constructs are non-essential, including scaffold/matrix attachment regions (S/MARs), enhancers, introns, tags and to some extent untranslated regions (UTRs). Each of these elements has implications in recombinant protein expression, by affecting either transcription, translation, stability or efficacy of purification. The main roles of each element are summarised in this figure and should be considered when designing RP expression constructs.

Recombinant protein expression can be optimised through increasing transcription and translation and decreasing mRNA and protein degradation. This section is dedicated to the transgene design considerations that can help improve *in planta* RP production (summarised in **Figure 1**).

### Improving transient transformation efficiency

Agroinfiltration with regular expression vectors only leads to localised RP production in tissues that have been directly infiltrated by the *Agrobacterium*, leading to patchy and variable expression. Transient transformation efficiency can be improved by using agroinfiltration with deconstructed vectors containing viral elements that allow the spread of the transgene throughout the plant *via* a process called magnifection, transiently transforming plant cells that usually would not be directly affected by agroinfiltration. These vectors have been extensively reviewed by Peyret and Lomonossoff (2015), but the most commonly used are the TMV-based vectors, using the magnICON system (Chen and Lai, 2015). Despite their frequent use, other viral vectors pose advantages, and should be used in two specific cases. The first is when non-tobacco plants are used, as magnICON vectors, being based on TMV, are only very efficient at transforming tobacco plants. In such cases, other vectors such as geminiviral vectors, which have a broad host range, may be more suitable for other plant species (Yamamoto et al., 2018). Secondly, the magICON vectors are unsuitable for producing proteins with heterosubunits, as multiple vectors have competing replicons, resulting in competing origins of replication, meaning cells will usually only amplify one of the vectors, producing only one of the subunits. Other vectors, including geminiviral vectors, with large copy numbers or without competing replicons are more suitable for co-expression of RPs, or expression of proteins with heterosubunits (Chen and Lai, 2015). Thus, the use of deconstructed viral vectors is often favourable to improve RP expression within transiently transformed plants, but these should be chosen on a case-by-case basis depending on the plant species being transformed and the properties of the RP. Improvements in transient transformation efficiency have also been reported in *N. benthamiana* plants by Norkunas et al. (2018) who used a modified infiltration buffer, a heat-shock of transformed plants, and co-expression of several different proteins to increase recombinant protein yields. By combining different improvements, these researchers reported a 3.5-fold increase in GUS expression when compared to the already efficacious pEAQ-HT system.

### Transcription

Optimising transcription is an easy way to improve expression levels because it requires alteration of the genetic construct without editing the coding sequence, so the resulting RP is not compromised. *Cis*-acting DNA elements can be included in the constructs to improve transcriptional rates. Broadly, every expression construct needs a promoter and a terminator, both of which influence the efficacy of transcription. Promoter sequences have different ‘strengths’, which ultimately means that strong promoters recruit RNA polymerase II more efficiently than weaker ones and create more transcripts. Some promoters are inducible, which means transcription factors bind the DNA only in response to stimuli, and others are tissue specific, meaning specific transcription factors only bind in certain tissues of multicellular organisms (Beringer et al., 2017). Generally, RPs are aimed to be produced in the largest quantities possible, so typically constitutive, global promoters (ie. expressed in all tissues) are used, such as the 35S Cauliflower Mosaic Virus (CaMV) promoter. Promoters should be chosen based on the need for *in planta* tissue-specific expression of the RP (if using stable transformants), and only constitutive if the RP will not affect plant growth. Finally, an important note to mention when using promoters is transcriptional gene silencing. Plants methylate homologous promoters, causing transcriptional transgene silencing in cells with two copies of the same promoter (Rajeevkumar et al., 2015). This means that it is beneficial to select for plant transformants with a single transgene integration event, or use different promoters when expressing two transgenes in the same plant line. Similarly, expressing the same RP with multiple constructs using different promoters, known as promoter stacking, can dramatically improve yields by avoiding or reducing transcriptional gene silencing. Damaj et al. (2020) managed to stack several promoters in stably transformed sugarcane plants, increasing expression by 147-fold when using stacked promoters compared to a singular promoter. Finally, there has been some progress made in the development of synthetic promoters, reviewed recently by Ali and Kim (2019), where promoters are engineered by combining different *cis*-acting DNA elements, sometimes combining multiple promoters in tandem, for novel or enhanced functions. Some synthetic promoters boasted an improvement in protein expression of 25-fold when compared to the commonly used CaMV 35S promoter (Kumar et al., 2011). Thus, a promoter should be chosen based on desired strength and cell-type-specific expression, with synthetic promoters, dual promoters, and promoter stacking available to further improve RP expression.

Choosing a terminator is also important as they terminate transcription and determine transcript stability, influencing transcript copy number. Commonly used terminators are the CaMV 35S, nopaline synthase (NOS), octopine synthase (OCS) and Heat Shock Protein (HSP) terminators (Nagaya et al., 2010; Limkul et al., 2015; Diamos and Mason, 2018). A factor to consider is the use of tandem units of promoters and terminators, as both double promoters and double terminators have been shown to improve protein expression (Khvatkov et al., 2018; Yamamoto et al., 2018). Diamos and Mason (2018) extensively characterised a series of terminators, and matrix attachment regions (MARs) in both tobacco and lettuce plants, showing that the use of different double terminator combinations and the employment of a MAR increased GFP production by over 60-fold compared to constructs using a single NOS terminator and no MAR. Interestingly, this is one of few studies to show that MARs improve RP expression in transient transformants.

Other commonly used but non-essential elements are enhancers, introns, and matrix-attachment regions. Enhancers increase transcript numbers through binding other trans-acting proteins, creating an active-chromatin hub (Tippens et al., 2018). As such, enhancers also contribute to tissue-specific gene expression, determined by the expression of these trans-acting proteins. In plants, introns are often used to improve transgene stability (Bourdon et al., 2001), and many of these contain enhancers, increasing mRNA production by up to 10-fold through intron-mediated enhancement (Rose, 2008). When designing the transgene, cryptic splice sites of AU-rich regions should be avoided to prevent the plant from recognising an unintended intron, and any intended introns should have their 3D-structure analysed before construct creation to avoid hairpins that could create siRNAs to silence the transgene (Smith et al., 2000). Large scale analysis of moss genes and subsequent codon optimization to reduce mRNA splicing resulted in a dramatic 12-fold increase in recombinant protein accumulation and correct intracellular trafficking (Top et al., 2021). Matrix attachment regions bind to the nuclear matrix and consequently improve transcription of nuclear-transformants, so should be considered for stably transformed plants (Allen, 2008; Diamos and Mason, 2018). MARs have been used in stable rice transformants to improve expression 3-fold (Vain et al., 1999)

Post-transcriptional gene silencing (PTGS), also termed RNA interference (RNAi), involves siRNA targeting of mRNA transcripts and subsequent transcript degradation, which is thought to have evolved to protect plants from viral attacks (Zhang et al., 2015). Fortunately, viruses have also evolved a defence against siRNAs, by producing proteins that bind to the siRNAs, preventing the degradation of the target mRNAs. These PTGS inhibitory proteins can be co-expressed in plants to increase expression dramatically. Most often, the p19 PTGS inhibitor from tomato bush stunt virus is used (Kuo et al., 2013; Merlin et al., 2014; Dong et al., 2019; Buyel et al., 2021), but others such as the P24 PTGS inhibitor from grapevine leafroll-associated virus-2 (Mardanova et al., 2017), P0 from Polerovirus, P17 from Aureusvirus, or NSs from Tospovirus are also available, though Peyret et al. (2019) recently established that P19 is the most effective of these. Ideally, these should always be used when high expression of a RP is desired, in both stable and transient transformants.

Finally, transcription is affected by position effects in stable transformants (Chen and Lai, 2015). This is because certain regions of chromatin are more active and euchromatic than others, and lengthy screening processes of many transformants are needed to isolate the best integrant lines. Fortunately, there are gene-targeting tools available for use in plants, including ZFNs, TALENs, and CRISPR variants (Sanagala et al., 2017).

### Translation

Translation efficiency can be improved by incorporating certain 5’-UTRs into the transformation construct, including the 5’-UTRs from alfalfa mosaic virus (AMV), which is reported to increase the production of the target protein by up to 4-fold (Mardanova et al., 2017). In fact, one system has developed into a commercial biotechnology product known as HyperTrans. This uses a modified 5’-UTR from cowpea mosaic virus (CPMV), with the deletion of an endogenous in-frame start codon in the UTR, enabling protein accumulation up to 20% of TSP (Sainsbury and Lomonossoff, 2008). These have been developed into commercially viable expression vectors that allow the expression of different transgenes at high levels (Sainsbury et al., 2009). The technology is used commercially for RP production. In fact, engineering efforts have recently been made to optimise both 5’ and 3’ UTRs through rational design; whilst the native CPMV 3’ UTR was not able to be improved, a synthetic 5’ UTR was developed that, when used in conjunction with the 3’ UTR, demonstrated double the protein expression of the HyperTrans system (Peyret et al., 2019).

Additionally, codon optimisation can increase translation efficiency by utilising the codons that are more commonly used in the host plants endogenous proteins (Webster et al., 2016; Buyel et al., 2021). This influences translation as tRNAs and their corresponding codons are in proportionate use (Plotkin and Kudla, 2010). In other words, codons that are rarely used by the host have fewer tRNAs in the cell, which can be a rate limiting step for protein production. One study found that codon optimisation of a Bt toxin, cryIA, increased protein accumulation by 100-fold through only changing codons by 21% (Perlak et al., 1991). Another study found that codon optimisation of miraculin increased protein accumulation by nearly 1.5-fold (Hiwasa-Tanase et al., 2010). A third study found that codon optimisation of the *S. cerevisiae* phytase enzyme, when expressed in rice, improved expression by nearly 120-fold, though a signal sequence was also added (Hamada et al., 2004). Thus, codon optimisation should be performed to improve translation efficiency for highly expressed proteins, however the secondary structure of the resulting mRNA should be computationally analysed for hairpin structures in case the modified codon would create siRNAs against the transgene and cause PTGS as mentioned above.

### Intracellular localisation, degradation and storage

Varying the eventual subcellular localisation of the RP can have implications on yield, stability, and purification. It is possible to sequester proteins into the nucleus, ER, vacuoles, mitochondria, plastids, or secrete them for the cell entirely (Drakakaki et al., 2006). For some proteins, such as Norwalk virus-like particles (NVLP) in *N. benthamiana* plants, cytosolic targeting (without the addition of any motifs) had the highest yield (Merlin et al., 2014). For others, such as hIL-6, endoplasmic reticulum (ER) targeted RP had expression up to 10-fold higher than apoplast and vacuole-targeted RP (Merlin et al., 2014). Importantly, intracellular localisation can be tissue dependent (Drakakaki et al., 2006), with reported differences between seeds and leaves (Hood et al., 2007), so this should be carefully investigated when using stable nuclear transformants in conjunction with tissue-specific promoter elements. The most common compartment to direct RPs is the ER, using KDEL/HDEL retention signals, typically chosen as the ER allows RPs to be stored in a less harsh environment with few proteases, and can result in antibody levels up to 100-fold higher than those expressed in the apoplast or cytosol (Fischer et al., 1999). However, ER retention causes RPs to avoid the Golgi, which means it is unsuitable for proteins which need downstream glycosylation (Villarejo et al., 2005; Egelkrout et al., 2012). Fortunately, multiple constructs can be created and co-expressed with different localisation sequences which can enhance RP production. Recombinant xylanase targeted to both the peroxisome and chloroplasts improved expression by 1.6-fold and 2.4-fold relative to only the chloroplast or peroxisomes respectively, despite similar levels of transcription (Hyunjong et al., 2005). Finally, some tags such as oleosin can be used to influence localisation and purification; these will be discussed more extensively in section 3.0.

Recombinant protein expression can also be optimised by reducing degradation, which can be done by protecting the protein, inhibiting proteases, or increase the protein’s stability. Tissue-specific or intracellular localisation influences protein stability. Some tissues, such as seeds, have naturally lower protease activity than other tissues and have intracellular storage vesicles (Stöger et al., 2000). This makes them ideal for RP expression when the RP needs to be stored. Alternatively, if the RP is not produced in tissue with naturally low protease activity, and is cytoplasmic, protease inhibitors can be co-expressed (Clemente et al., 2019), or the expression of endogenous proteases can be knocked-down (Kuo et al., 2013). Additionally, intracellular compartmentalisation of RPs can reduce degradation and improve stability.

Importantly, researchers studying roots and cell cultures have found that protein degradation is largely dependent on the expression system, with rhizosecreted proteins experiencing more peptidase activity than proteins secreted into culture medium; further investigation determined that these are largely due to serine and metallo-peptidases (Lallemand et al., 2015). Thus, knocking down expression of these may be considered to reduce recombinant protein degradation. Furthermore, Niemer et al. (2014) found that different recombinant monoclonal antibodies have different susceptibilities to serine- and cysteine-protease degradation, dependent on internal cleavage sites within a recombinant protein, demonstrating the importance of the amino acid sequence of expressed proteins.

Strategies to reduce endogenous plant protease activity have been reviewed by Mandal et al. (2016). The addition of stabilizing agents such as gelatin or polyvinylpyrrolidone (Wongsamuth and Doran, 1997; Häkkinen et al., 2014) can improve stability. Alternatively, co-expression of protease inhibitors such as tomato SlCDI (Goulet et al., 2012) or SlCYS8 (Robert et al., 2013) can reduce degradation, though this has been shown to be dependent on leaf age in transgenic tobacco (Robert et al., 2013). A more recent study by Grosse-Holz et al. (2018) identified three novel protease inhibitors, PR4 and Pot1 from *N. benthamiana*, and human TIMP, inhibitors of cysteine, serine, and metalloproteases, respectively. The researchers expressed recombinant α-Galactosidase, erythropoietin, and IgG antibody VRC01, and co-expression of each protease inhibitor showed similar improvements in each recombinant protein, between 2- and 27-fold, demonstrating the power of protease inhibition. Similarly, in moss, Hoernstein et al. (2018) identified four serpin proteins that irreversibly inhibit proteases, which may present promising targets to further improve RP stability. Targeted knockdown of genes can reduce degradation as demonstrated by RNAi-mediated knockdown of cysteine protease Rep-1 (Kim et al., 2008) in rice suspension cultures when producing recombinant human proteins. More broadly, aspartic-, cysteine-, metallo- and serine-proteases have been simultaneously knocked down by RNAi to improve RP production in tobacco BY-2 cell cultures (Mandal et al., 2014). Similarly, in moss, a subtilisin family protease was knocked out to improve early production of recombinant human α-Galactosidase A (Hoernstein et al., 2018). Finally, some fusion proteins can increase RP stability *in planta*, including elastin-like peptides (Conley et al., 2009; Kaldis et al., 2013), zein-derived peptides (Torrent et al., 2009; Joseph et al., 2012), and hydrophobins (Reuter et al., 2014), which largely stabilise the protein through directing the RP to storage structures. The expression of GFP fused to hydrophobin improved protein levels by 2-fold in both stably and transiently transformed *Nicotiana* plants, and by 3-fold in tobacco BY-2 cell cultures (Joensuu et al., 2010; Gutiérrez et al., 2013; Reuter et al., 2014). However, some proteins, such as β-glucuronidase, are naturally more stable in plant cells than others (Kozlov et al., 2019), so whether a stabilising tag is necessary should be decided based on the specific stability of the expressed RP. Tags should be more carefully considered for purification purposes, discussed in the following section.

## Tags and purification

Recombinant protein purification is considered the costliest stage in plant RP production, with comparable costs to other expression systems (Kuo et al., 2013; Merlin et al., 2014; Chen and Davis, 2016; 2016; Dong et al., 2019). Extraction of plant proteins has been reviewed by Wilken and Nikolov (2012) with some further developments in physical (Buyel and Fischer, 2015) and chemical extraction (Dong et al., 2019). Plant protein purification often relies on chromatography techniques, although non-chromatographic techniques also exist and have shown success in plants. These include flocculation (Elmer et al., 2009; Buyel and Fischer, 2013; Buyel and Fischer, 2014), ultrafiltration and diafiltration (Buyel and Fischer, 2013; Opdensteinen et al., 2019), heat-precipitation (Menzel et al., 2016; Opdensteinen et al., 2020), and pH-precipitation (Menzel et al., 2016; Opdensteinen et al., 2020), with several methods reviewed by Buyel (2022). These will not be discussed in detail in this section, which will instead focus on the use of purification tags in plants as they are encoded in DNA and are often designed with the expression construct.

The use of affinity tags in RP production has been recently reviewed (Mahmoodi et al., 2019), with a diverse array of tags to choose from. There are many existing examples of affinity tag usage for plant RP purification. Antibody production usually utilises protein A affinity chromatography and does not require an additional fusion tag and is an efficient but expensive process that is only applicable to antibodies (Merlin et al., 2014). As a result, a dual-purification technique was developed by Jugler et al. (2020), which utilised a hydrophobin fusion to protein A (HPA). When the HPA fusion protein was mixed with the monoclonal antibody (mAb), two-phase separation could be performed to isolate the mAb from the bulk of endogenous proteins, which are retained in the aqueous phase. Following this, the addition of a low pH buffer can separate the mAb from the HPA fusion protein, allowing high purity and yield mAb recovery. This method is particularly interesting as it utilises a fusion-protein that does not involve the RP (mAb) of interest. However, despite this methods success, due to the use of protein A affinity purification, this protocol is still only applicable to antibodies.

Consequently, most affinity tags used in plant RP purification use fusions to the RP itself. One study utilised Arabidopsis to produce a recombinant membrane protein, STT3a, and used tandem affinity purification of a FSG tag (comprised of three FLAG–streptavidin binding peptide, a TEV protease cleavage site, and two protein G domains) and retrieved the RP following TEV-mediated cleavage with high enough yield and purity for rough structural studies at resolutions of 30Å (Jeong et al., 2018). A cheaper method is the use of IgG-affinity columns to purify proteins with a *Staphylococcus aureus* Z-tag. This has been used to produce chloroplast-expressed hIGF, at a cleavage efficiency of only 40%, but using hydroxylamine to cleave the tag (Daniell et al., 2009). Histidine residues have an affinity to nickel beads, so His-tags or proteins containing histidine residues in tandem are also used for purification using immobilised metal affinity chromatography (Wilken and Nikolov, 2012). However, despite their common use, His-tags are not always specific, with some endogenous plant proteins containing natural histidine motifs, so one group developed a hybrid tag, termed ‘Cysta-tag’, comprised of the His-tag with an additional multicystatin domain SlCYS8, which enhances RP levels in tobacco, which enabled a tagged RP to have 25% yield recovery and 90% purity (Sainsbury et al., 2016). Other researchers have created hybrid tags containing several different protein domains. Qi and Katagiri (2009) created a purification tag, called the HPB tag, which utilises a biotin carboxyl carrier protein domain (BCCD) derived from the *Arabidopsis* 3-methylcrotonal CoA carboxylase, and enables purification of low abundance membrane protein complexes using streptavidin beads, and subsequent cleavage of the tag using PreScission protease, leaving only a single short copy of the hemagglutinin (HA) tag for further purification if desired.

Alternatively, tags have been developed that do not rely on chromatography methods, including oleosin, elastin-like proteins (ELPs) and γ-Zein-derived peptides. Oleosin is similar to hydrophobin proteins, as when fused to a RP oleosin causes it to become hydrophobic and localise to the lipid-rich layer of a cell, allowing separation from the bulk of intracellular proteins through centrifugation (Wilken and Nikolov, 2012; Gerszberg and Hnatuszko-Konka, 2022). Parmenter et al. (1995) managed to purify recombinant aprotinin from maize seeds using an oleosin tag, with yield recovery of 49% but relatively low purity of 79%. ELPs allow similar purification, by causing the RP to become insoluble at raised temperatures, allowing easy separation from endogenous proteins (Rigano et al., 2013). Finally, γ-Zein-derived peptides cause localisation into the ER, and allow purification through differential centrifugation (Torrent et al., 2009; Joseph et al., 2012).

The disadvantage of affinity tags is that they sometimes must be cleaved to ensure normal RP function, a step that is usually inefficient and reduces RP yields further. Cleavage mechanisms are also highly varied, with many options available. Often, protease-mediated cleavage is used, where the protein coding sequence incorporates a protease recognition site between the RP and the tag. TEV protease is a widely used and well characterised protease, but several others are available including endoproteases such as factor Xa, enteropeptidase, thrombin and 3C protease, and exoproteases such as aminopeptidases and carboxypeptidases, though exoproteases are rarely used for RP production (Waugh, 2011). Frey & Görlich (2014a) discuss a series of alternative proteases with reportedly improved functions, including SUMO-specific SENP1 and NEDD8-specific proteases from *Brachypodium distachyon*, NEDP1 protease from *Salmo salar*, Atg4p from *Saccharomyces cerevisiae* and Usp2 from *Xenopus laevis*. Of these, bdSENP1 showed 10,000-fold higher activity than TEV protease at 0-4°C. It also showed increased salt tolerance which is useful as extraction buffers often contain high salt-concentrations. In an accompanying paper, the researchers tested these proteases on several recombinant proteins (Frey & Görlich, 2014b). Similarly, inteins can be used to remove tags, and have been reviewed by Lahiry et al. (2017). These are protein sequences that, under the correct environmental conditions, can ligate flanking peptides called exteins, excising themselves in a process termed protein splicing. Intein activity can be regulated by pH, temperature, the presence of reducing agents, salts, small molecules, and light (Lahiry et al., 2017). However, limitations of these include premature cleavage, product losses, the potential need for unfavourable reducing agents, and varying suitability depending on the amino acid sequence surrounding the exteins. A successful example comes from Islam et al. (2018) who developed a plant-based affinity tag with a family-3 cellulose-binding domain (CBD), CBM3, which binds microcrystalline cellulose (MCC). This was used in conjunction with bdSENP1 which enabled efficient tag cleavage following purification. This method is cheap due to the low cost and high availability of cellulose for purification. One potential disadvantage that the researchers acknowledged was the large size of the CBD (18kDa), which may affect the function and folding of the fused RP. The researchers demonstrated that the CBD tightly bound MCC, allowing RPs to be purified at over 95% purity in a single step. The researchers also noted that traditionally used proteases, including TEV, are expensive, and intein-based cleavage was inefficient and time consuming, requiring high concentrations of potentially damaging reducing agents to activate. Islam et al. (2018) utilised the SUMO-based cleavage as there is no additional overhanging sequence left following cleavage, and the process is highly efficient.

To summarise, protein tags substantially aid purification efficiency, but at the cost of yield due to multi-step purification processes. Thus, purification without the use of tags is often desirable. mAbs can be produced in plants rapidly, cost-effectively, and with high yields (Diamos et al., 2020). For non-antibody proteins, tags are often directly fused to the RP, and must be cleaved. Fortunately, cheaper binding partners are now available and more efficient tags have recently been developed allowing some flexibility when deciding on purification methods. Ultimately, tag usage should also be chosen on a case-by-case basis, paying careful attention to the properties of the RP in different conditions. **Table 3** summarises the purification tags discussed. A non-plant-specific table is available in a review by Kimple et al. (2013).

**Table 3:** Summary of the protein purification tags discussed in this review.

Tag	Purification Method	Notable Addition(s)	Reference
CBM3	Microcrystalline cellulase affinity column	Components used are extremely cheap and available Protein tag is very large at 18kDa and may affect structure Used in conjunction with SUMO cleavage leaving no additional overhang	(Islam et al., 2018)
Cysta-tag	Utilises immobilised metal affinity chromatography (IMAC)	His-tag with additional SlCYS8 domain Improved stability with one-step purification	(Sainsbury et al., 2016)
Elastin-like peptides	Heat-mediated purification	ELPs make the RP insoluble at raised temperatures	(Rigano et al., 2013)
FSG tag	Tandem affinity purification	Purified by protein G domains	(Jeong et al., 2018)
Glutathione S-transferase (GST)	Glutathione	Commonly used but not suitable for every RP	(Kimple et al., 2013)
His-tag	IMAC using nickel beads	Purifies endogenous plant proteins containing His-residues in tandem	(Wilken and Nikolov, 2012)
HPB-tag	Streptavidin pulldown, cleavage and subsequent HA-tag purification if desired	Synthetically designed tag able to purify low-abundance membrane protein complexes	Qi and Katagiri (2009)
Hydrophobin	Aqueous two phase partitioning followed by protein A affinity chromatography	Hydrophobin is fused to Protein A not to the recombinant antibody Only applicable to antibodies	(Jugler et al., 2020)
None – utilise RP	Immunoprecipitation	When no tag is used, and antibodies are raised against the RP itself The generation of specific antibodies is expensive No cleavage of tags is needed Miniaturisation attempts made to improve efficacy	(Sandison et al., 2010)
None – utilises antibody	Protein A affinity chromatography	Only applicable to antibodies but is widely used	(Merlin et al., 2014)
Oleosin	Two-phase separation where RP enters lipid phase, and can be cleaved to separate it	Makes RP lipophilic until the oleosin tag is cleaved	(Wilken and Nikolov, 2012)
Z-tag	Staphylococcal protein A affinity chromatography	Purified a chloroplast-expressed RP Only applicable to immunoglobulins	(Daniell et al., 2009)
γ-Zein-derived peptides	Cause ER localisation allowing protein to be separated by centrifugation	Similar to localisation motifs such as KDEL	(Joseph et al., 2012).

This table is not an exhaustive list, but shows a range of examples of purification tags and their respective methods that are available to researchers.

## Conclusion

**Figure 2:**
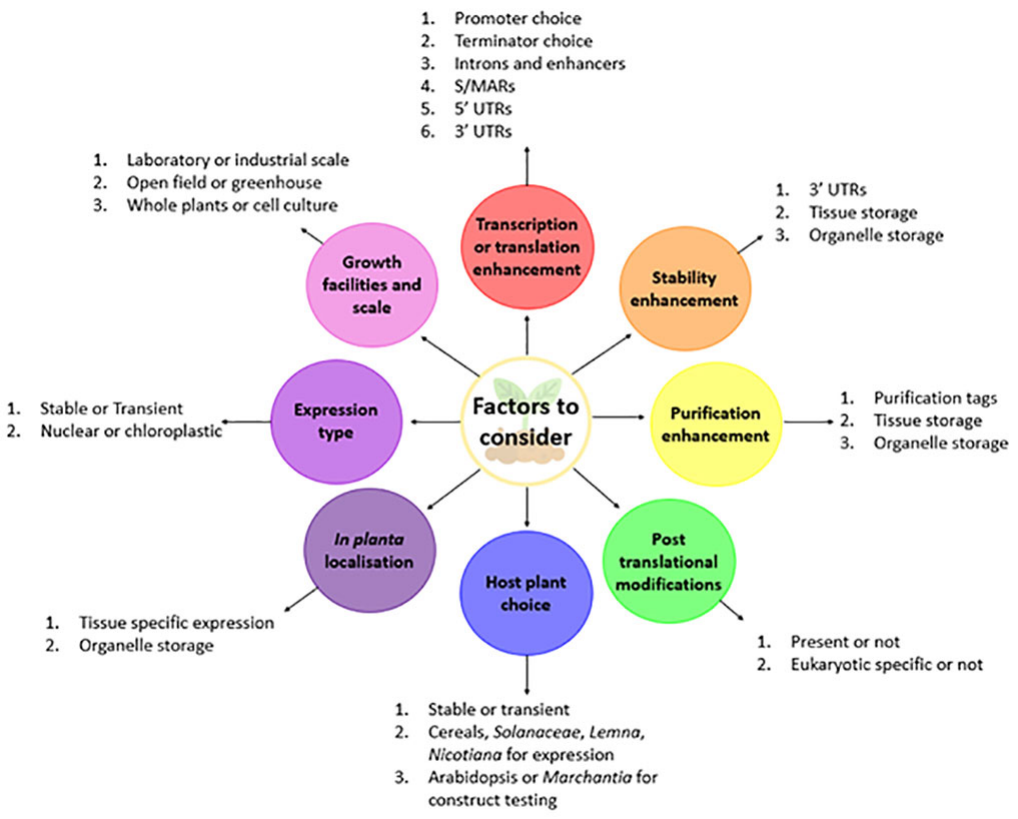
Summary of factors to consider when expressing recombinant proteins in plants.

Overall, plants constitute a promising recombinant protein production platform, with several unique advantages that may make them specialists at producing recombinant proteins. When producing RPs, plants give researchers large amounts of flexibility, in that several different modes of transgenesis can be used, whether this is stable or transient, nuclear or chloroplastic, each with their own unique advantages that can cover the breadth of requirements for any recombinant protein. This flexibility results in a series of co-dependent decisions that a researcher can make when expressing RPs in plants, summarised in **Figure 2**. It is possible that plant-based protein production platforms could become cheaper than prokaryotic systems, as downstream processing methods begin to rely on cheaper reagents which have been developed in recent years. While plants are able to produce nearly any protein, it is most likely they will only be the dominant production system for proteins that have complex post-translational modifications and are required rapidly. To some degree, this is already realised as they are emerging to be the dominant antibody production platform, with pharmaceuticals not far behind, due to their unique advantage that RP-containing tissue can be consumed. Fortunately, recent and seemingly continuous developments in plant RP production and purification are rapidly advancing the field, with researchers now able to dramatically improve transcriptional and translational efficacy, maximise the number of transformed plant cells, and extract RPs with higher yields and purities than before. Consequently, due to these advancements, extremely cheap upstream costs, unparalleled scalability, and relative lack of specialist equipment, it would be unsurprising if plants eventually became the dominant protein production platform worldwide.

## Author contributions

All authors listed have made a substantial, direct and intellectual contribution to the work, and approved it for publication. RC generated the figures, tables and led the writing.
